# NMDARs Translate Sequential Temporal Information into Spatial Maps

**DOI:** 10.1016/j.isci.2020.101130

**Published:** 2020-05-01

**Authors:** Masaki Hiramoto, Hollis T. Cline

**Affiliations:** 1 The Dorris Neuroscience Center, Department of Neuroscience, The Scripps Research Institute, 10550 North Torrey Pines Road, La Jolla, CA 92037, USA

**Keywords:** Developmental Neuroscience, Sensory Neuroscience, Techniques in Neuroscience

## Abstract

Spatial representations of the sensory world are important for brain function. Timing is an essential component of sensory information. Many brain circuits transform the temporal sequence of input activity into spatial maps; however, the mechanisms underlying this transformation are unclear. Different *N*-methyl-D-aspartate receptor (NMDAR) response magnitudes result in synaptic potentiation or depression. We asked whether NMDAR response magnitude also affects the transformation of temporal information into directional spatial maps. We quantified retinotectal axon branch dynamics in *Xenopus* optic tectum in response to temporal sequences of visual stimulation. Reducing NMDAR responses by 50% inverts the spatial distribution of branch dynamics along the rostrocaudal axis in response to temporal patterns of input, suggesting that the magnitude of NMDAR signaling encodes the temporal sequence of inputs and translates the temporal code into a directional spatial map using structural plasticity-based branch dynamics. We discuss how this NMDAR-dependent decoding mechanism retrieves spatial information from sequential afferent activity.

## Highlights

• NMDAR response magnitude encodes the temporal sequence of inputs
• NMDAR mechanism decodes spatial information from sequential input activity
• NMDAR attenuation inverts the temporal to spatial transformation
• NMDAR activity alters the spatial distribution of dynamic and stable branches

## Graphical Abstract

**Figure:**
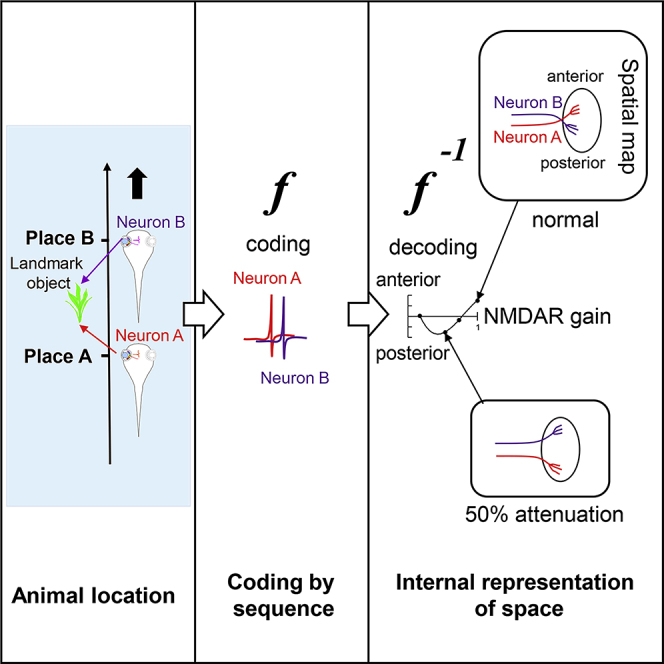

## Introduction

Timing is an essential component of information in the brain. Many brain circuits transform the temporal sequence of input activity into spatial maps of inputs as seen in sensory circuits and circuits for cognition, such as entorhinal cortex (Hafting et al., 2005, Moser et al., 2017) and cerebellum (Kitazawa et al., 1998). Spatial sensory maps thereby recapitulate the spatial distribution and timing of sensory input activity. Similarly, place fields in the CA1 region of hippocampus are organized to reflect a spatial map of the environment that animals have recently experienced in the temporal domain (Buzsaki and Tingley, 2018, O'Keefe, 1999). The topographic organization of sensory maps is essential for information processing (Petersen, 2019, Schreiner et al., 2000, Sperry, 1943), sensory motor transformations (Knudsen, 2002, Sperry, 1943), navigating through the environment (Lu et al., 2015), memory-based and predictive behaviors, as well as cognitive functions (Ghahramani and Wolpert, 1997, Graziano and Aflalo, 2007). Importantly, maps are continuously updated by sensory experience, likely by spatial information encoded in the patterns of activity (Cline, 1998). Although temporal activity patterns are known to contain rich spatial information (Buzsaki and Tingley, 2018), the mechanisms that transform the temporal pattern of activity into spatial maps are unclear.

Experience-dependent plasticity of the visuotopic map in *Xenopus* optic tectum provides an excellent system to analyze how temporal information is transformed into spatial information. The visuotopic map in the tadpole tectum is continually updated by visual input activity to accommodate developmental changes in the size of the tectum and the size and position of the eyes (Cline, 1998, Cline and Constantine-Paton, 1989, Reh and Constantine-Paton, 1984). With forward-directed navigation and optic flow, objects move along the anterior to posterior axis of the tadpole's visual field and sequentially activate retinal ganglion cells (RGCs) from temporal to nasal retinal positions, transforming the spatial order of visual stimuli into a temporal sequence of RGC activity. The temporal sequence of RGC activity stimulated by optic flow is then transformed into the spatial distribution of RGC axons along the rostrocaudal axis in the target optic tectum (Hiramoto and Cline, 2014). Furthermore, reversing optic flow and therefore the sequential activity patterns in RGCs disrupted the organization of axon arbors in the tectum. Experience-dependent plasticity of the visuotopic map is an example of a transformation of spatial visual information into temporal information in RGC activity followed by a transformation of the temporal information in RGC activity into the spatial distribution of retinotectal axons, or a spatial to temporal to spatial (STS) transformation. In this way, this experimental system enables analysis of how temporal information is transformed into spatial information, using RGC axon projections as a readout.

*N*-methyl-D-aspartate receptors (NMDARs) are highly conserved voltage-dependent calcium-permeable ionotropic glutamate receptors that function in diverse plasticity mechanisms across species (Bear, 1996, Constantine-Paton, 1990, Feldman, 2012, Schiller et al., 2018). NMDARs are required for synaptic and structural plasticity throughout the brain (Cline et al., 1987, Espinosa and Stryker, 2012, Hamodi et al., 2016, Hiramoto and Cline, 2014, Iwasato et al., 2000, Kirkby et al., 2013, Kleinschmidt et al., 1987, Mizuno et al., 2014, Munz et al., 2014, Ruthazer et al., 2003, Yang et al., 2018). Recent reports suggest that NMDARs have additional functions integrating temporal information. For instance, forebrain NMDAR knockout impairs oscillatory activity that encodes a sequence of locations and impairs memorizing the sequence of locations (Cabral et al., 2014). NMDARs also mediate the formation of CA1 place fields by integrating inputs over a time frame relevant to spatial navigation behaviors (Bittner et al., 2017). These studies suggest that NMDARs extract information about the spatial sequence of locations from the temporal sequence of afferent activity. In retinotectal map plasticity, NMDARs are required for STS, in which the temporal sequence of RGC activity is transformed into the directional organization of the spatial map along the rostrocaudal axis of the tectum (Hiramoto and Cline, 2014). This was unexpected because in topographic map plasticity NMDARs are thought to strengthen and stabilize coactive convergent inputs and to weaken and destabilize uncorrelated inputs, mechanisms that are independent of the temporal sequence of inputs and that do not impart directional information to the topographic map (Cang et al., 2018, Constantine-Paton, 1990, Espinosa and Stryker, 2012, Lim et al., 2008, Munz et al., 2014).

Here, we explored how NMDARs transform the temporal sequence of convergent input activity into a directional component of the spatial map. In particular, we were interested in testing whether the magnitude of the NMDAR response would affect the directional shift in dynamics of axon branches along the rostrocaudal axis of the tectum, analogous to the effect of NMDAR response amplitude on the sign of synaptic plasticity, seen as synaptic depression or potentiation (Bear et al., 1987, Bear and Malenka, 1994, Feldman, 2012, Kirkwood and Bear, 1995, Shouval et al., 2002). We analyzed the structural plasticity of retinotectal axon arbors induced by temporal sequences of RGC activity in the *Xenopus* tadpole visual system and tested whether the amplitude of NMDAR responses regulates the transformation of the temporal sequence of information into directional spatial information. *In vivo* time-lapse analysis of axon branch dynamics demonstrated that attenuating NMDAR activity by 50% inverted the directional growth of axons in response to temporal sequences of visual input, suggesting that the directionality of the temporal to spatial transformation in the STS rule is mediated by NMDAR response amplitude. Furthermore, decreasing NMDAR activity in one of the two convergent pathways using the use-dependent blocker MK801 was sufficient to shift the spatial distribution of retinal inputs. Together, these data indicate that the magnitude of NMDAR signaling encodes the temporal sequence of afferent inputs and translates the temporal code into a spatial representation of RGC activity, which in turn represents the location of moving objects in visual space.

## Results

### The Temporal Sequence of Afferent Activity Is Transformed into the Spatial Order of Projections

**Figure 1:**
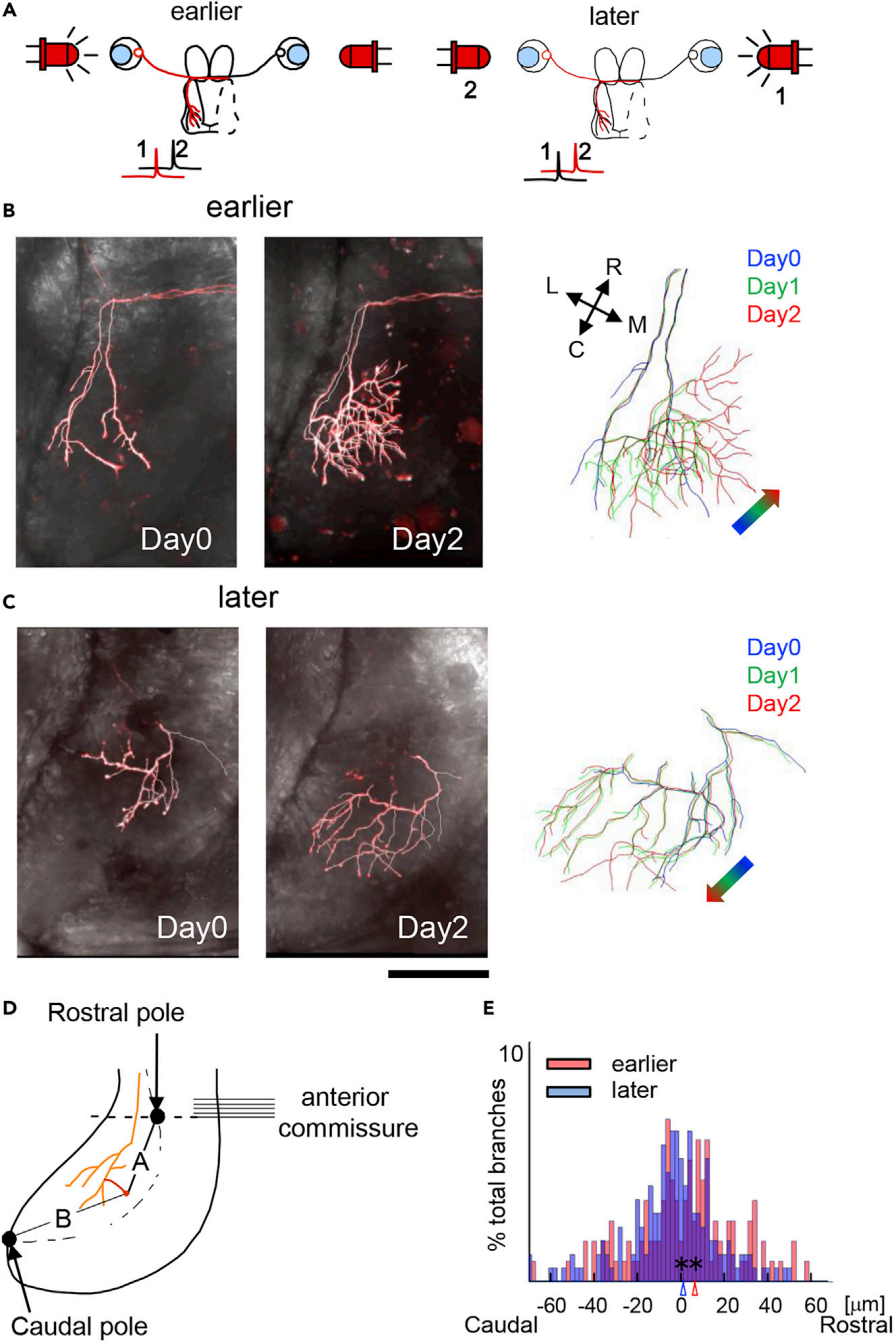
The Temporal Sequence of Afferent Activity Is Transformed into the Spatial Order of Axon Projections (A) Experimental design to assay the transformation of the temporal sequence of stimulation into a spatial representation of sequential axon activity. One tectal lobe is ablated so that the RGC axons from both eyes project to the remaining tectum. By stimulating the eyes sequentially with LEDs, the two groups of the convergent RGC axons are activated in a sequence. RGCs in the left eye are sparsely labeled with tdTomato (red) for *in vivo* time-lapse imaging of dynamic changes in axon arbor morphology and analysis of changes in branch tip positions. The sequence of activity is schematized under each panel. Left panel: The left eye with the labeled axons is stimulated 15 ms earlier (dt = −15ms) than the right eye. Right panel: The left eye is stimulated 15 ms later (dt = +15ms) than the right eye. The animals are raised in dark until the stimulation protocol begins. The eyes are stimulated for 10 h per day starting after the images were collected on Day 0. (B and C) Examples of the directional axon morphology branch tip shift with opposite temporal sequences of retinal activity. The left eye was stimulated 15 ms earlier or later than the right eye, as schematized in (A), for earlier or later stimulation of the eye with the labeled axons, respectively. Left: Time-lapse confocal images of z series through axons imaged before and 2 days after the stimulus protocol, superimposed on differential interference contrast images of the tectum. Right: Axon reconstructions from images collected on Day 0 (blue), Day 1 (green), and Day 2 (red) are superimposed. The colored arrow shows the overall direction of branch shift over the 3 days of imaging. The rostrocaudal (R<->C) and mediolateral (M<->L) orientation of the tectum is shown in the inset. (B and C) show 2 and 1 axons, respectively. (D) Quantification of branch tip movement. The relative distance from each axon branch tip (A) to the rostral and the caudal poles (B) was measured for each time point. Changes in the relative positions between time points were determined and expressed as a shift toward the rostral or caudal pole. (E) Histogram of the proportion of total branch tips that shift toward the rostral or caudal poles for axons stimulated earlier (red) or later (blue), where 0 indicates no shift. Bin = 1.5 μm. Bootstrap (N = 10,000) was used to determine the significance (p = 0.0013) of the difference in the mean values. Scale bar, 100 μm in (B, C: left) and 67 μm in (B, C: right). N = 144 and 219 branches in 7 and 7 animals for earlier and later conditions, respectively.

In the tadpole visual system, RGC axons from each eye innervate the contralateral optic tectum. We generated a binocular retinotectal circuit by ablating one tectal lobe, forcing axons from both eyes to converge on the remaining tectal lobe (Ruthazer et al., 2003, Straznicky and Glastonbury, 1979). We used this system to visualize *in vivo* branch tip rearrangements in response to visual stimulation provided sequentially to the two eyes with a controlled temporal offset. Animals were held in a chamber with light-emitting diodes (LEDs) positioned to stimulate both eyes sequentially with a 15-ms interval between the left and right eyes at 11 Hz. We choose the 15-ms interval because sequential stimuli with a 15- to 20-ms interval induced the most consistent branch tip shifts in our previous study (Hiramoto and Cline, 2014). The shift in branch tip positions was less at a 50-ms interval and was not detected at an interval of 100 ms. The stimulation continued for 10 h per day for 2 days, which was shown previously to induce directed branch tip rearrangements (Hiramoto and Cline, 2014). We labeled single or a few RGC axons in one eye and stimulated the labeled eye either 15 ms later or 15 ms earlier than the other eye, referred to as dt = +15 ms or dt = −15 ms, respectively (Figure 1A). Labeled axons were imaged before stimulation started (Day 0) and after 2 days of visual stimulation (Day 2). We quantified the structural rearrangements of axon branches by calculating the shift in position of each branch relative to reference points at the rostral and caudal poles of the tectum (Figure 1D), similar to Hiramoto and Cline (2011). We focused on relative changes in the branch tip positions to normalize the influence of activity-independent factors. When the eyes were stimulated in sequence to produce sequential firing in their axons, *in vivo* time-lapse imaging demonstrated that the labeled axon branches shift their position along the rostrocaudal axis of the optic tectum such that the input that fires earlier than convergent inputs from the other eye shifts branches toward relatively more rostral tectal positions and the input that fires later shifts branches caudally (Figures 1B, 1C, and S1A), indicating that the shift increases with longer stimulation periods. The Wald-Wolfowitz runs test demonstrated that the directional changes of individual branches within an axon were independent of one another (Table S1). The changes in position of each branch tip along the rostrocaudal tectal axis over 2 days are displayed in a histogram (Figure 1E), with axons stimulated earlier than convergent inputs in red and those stimulated later in blue. The peaks in the distributions of branch movements for earlier stimulated (red) and later stimulated (blue) axons, marked by the red and blue carrots on the x axis, were significantly different between the two groups of axons (p = 0.013, N = 10,000 bootstrap). This analysis demonstrated that the temporal sequence of convergent afferent activity directed the spatial distribution of axon branches in the tectum, recapitulating the rostrocaudal distribution of retinal inputs that occurs in response to anterior-to-posterior motion of visual stimuli.

### Attenuation of NMDAR Activity Flips the Direction of Axon Branch Growth Induced by Sequential Afferent Activity

**Figure 2:**
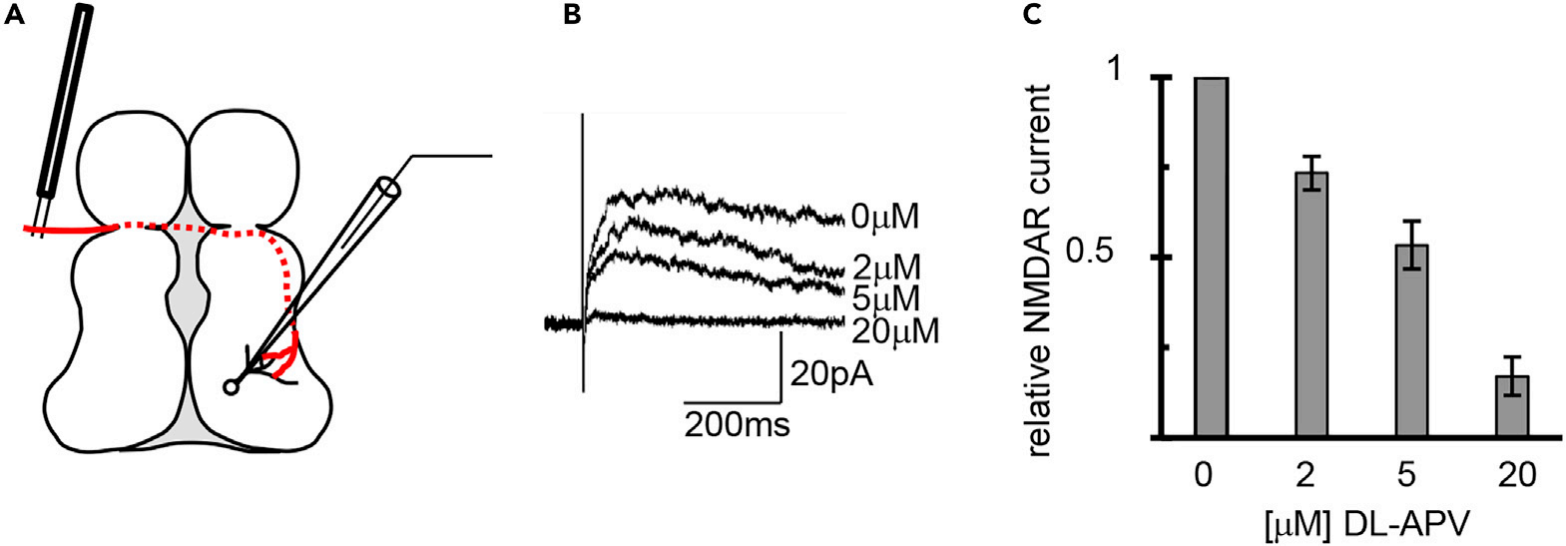
APV Dose-Dependent Block of NMDAR (A) Schematic of experiment: NMDAR currents in response to stimulating RGC axons (red) in the optic tract were recorded in whole-cell mode from tectal cells held at +40 mV holding potential in the presence of 20 μM NBQX and 100 μM picrotoxin. (B) Averages of 20 traces of NMDAR-mediated excitatory postsynaptic currents recorded in the presence of increasing concentrations of DL-APV added to the bath. (C) Relative total charge transfer normalized to 0 μM APV. 0 μM: 100%, 2 μM: 73.4% ± 10.4%, 5 μM: 53.4% ± 11.5%, 20 μM: 17.0% ± 7.5%. N = 5.

**Figure 3:**
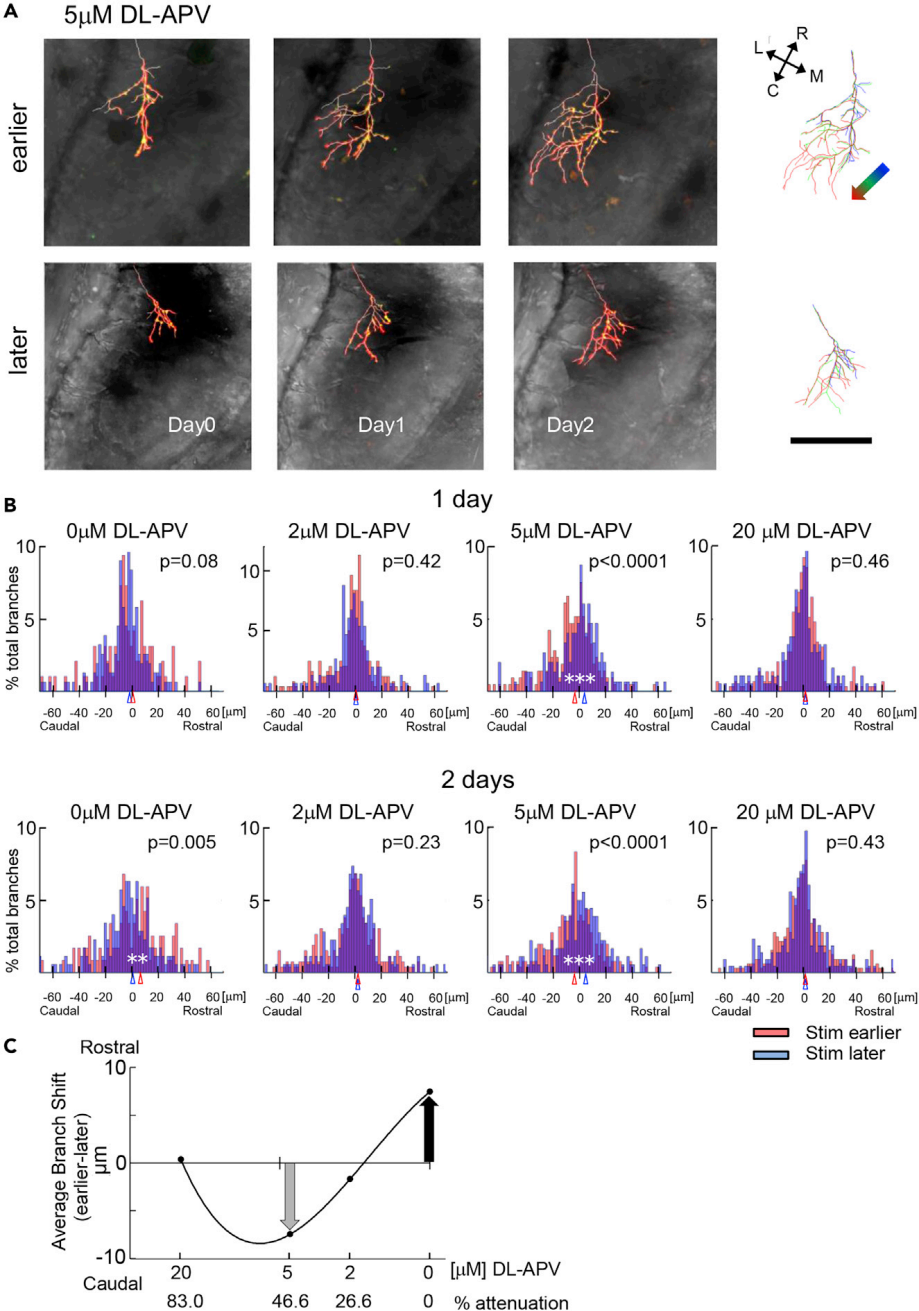
Attenuation of NMDAR Activity Flips the Direction of Axon Branch Growth Induced by Sequential Afferent Activity (A) Examples of effect of 5 μM APV on directional axon morphology branch shift when left and right eyes were stimulated in sequence, as in Figure 1A. Single axons in the eye ipsilateral to the tectum were labeled. The labels “earlier” and “later” refer to the ±15-ms offset in stimulation sequence of the imaged axons. Stimulation started after imaging at “day 0,” marked under the panel. Left: Time-lapse confocal images of z series through axons imaged before (day 0) and 1 and 2 days after the stimulus protocol, superimposed on differential interference contrast images of the tectum. Right: Axon reconstructions from images collected on Days 0 (blue), 1 (green), and 2 (red) are superimposed. The colored arrow in the upper row shows the overall direction of branch shift over the 3 days of imaging. The orientation of the tectum is shown in the inset. Data from other APV concentrations are shown in Figure S2. (B) Quantification of branch shift along the rostrocaudal axis (from Figure 1E). Histograms of the amplitudes of branch tip movement toward the rostral or caudal tectal poles for axons stimulated earlier (red) or later (blue). Bin = 1.5 μm. Bootstrap (N = 10,000) was used for statistics. p values are shown in the figure. N = 99 and 256 branches in 5 and 8 animals for earlier and later conditions, respectively. (C) Relationship between the relative NMDAR activity and the average relative branch tip shift. Scale bar, 100 μm in (A). The shift of the later group is subtracted from that of the earlier group.

We tested whether reducing NMDAR currents might interfere with transforming the temporal sequence of input activity into spatial information. To test if there is dose-dependent effect of NMDAR activation on the STS plasticity rule, we used amino-5-phosphonovaleric acid (DL-APV) to produce a graded decrease in the amplitude of NMDAR currents. We stimulated the optic nerve (Figure 2A) measured NMDAR-mediated excitatory postsynaptic currents in animals treated with 2, 5, and 20 μM DL-APV, recorded in the presence of 20 μM 2,3-Dioxo-6-nitro-1,2,3,4- tetrahydrobenzo[f]quinoxaline-7-sulfonamide (NBQX) and 100 μM picrotoxin at +40 mV holding potential. Measuring total change transfer from 20 to 30 ms after the onset of the NMDA current showed that 2, 5, and 20 μM DL-APV reduced NMDAR currents by 26.6%, 46.6%, and 83.0%, respectively (Figures 2B and 2C). We then exposed animals, prepared as described above, to a range of APV doses during 10 h of visual stimulation with dt = ±15-ms interstimulus interval (Figures 3A and S2). Labeled retinotectal axons were imaged three times, at daily intervals after the visual stimulation protocol. Changes in branch tip positions from Day 0 to Day 1 and Day 1 to Day 2 were analyzed and plotted as histograms showing the shift in branch position along the rostrocaudal tectal axis. In controls without APV, earlier-stimulated axon branch tips showed a significant spatial shift toward rostral tectum compared with later-stimulated axons (Figure 3B). This effect was blocked by 20 μM APV: neither earlier- nor later-stimulated axon branches showed a significant change in position over the 2 days of experiments (Figure 3B), demonstrating that this plasticity is NMDAR dependent. Axon branches in animals treated with 5 μM APV significantly shifted their positions in the optic tectum in response to visual stimulation; however, the direction of branch movement was opposite to that seen without APV. Earlier-stimulated axons shifted their branch tips toward more caudal tectal positions than later-stimulated axons (Figure 3B). This effect of 5 μM APV on branch dynamics was significant after the first 10-h visual stimulation period in APV. Treatment with 2 μM APV eliminated the directed branch tip movement, even though it inhibited NMDAR currents less than 5 μM APV. These data suggest that the magnitude of the NMDAR response encodes directionality of the branch tip movement in response to visual stimulation, and specifically that the direction of branch tip shift is reversed when NMDAR current is attenuated by greater than ~25% (Figure 3C).

### Graded NMDAR Function Alters the Spatial Distribution of Dynamic and Stable Branches

Changes in branch dynamics and stability contribute to experience-dependent structural plasticity in sensory inputs during map refinement. Correlated convergent input activity stabilizes axon branches, whereas inputs that are not correlated are destabilized (Ruthazer et al., 2003). Data indicating that NMDAR activity is required for both correlation-dependent branch stabilization and non-correlation-dependent branch retraction (Munz et al., 2014, Ruthazer et al., 2003, Witte et al., 1996) suggested that different magnitudes of NMDAR signaling could generate these opposing outcomes, analogous to postulated mechanisms underlying NMDAR-mediated increases and decreases in synaptic strength (Bear and Malenka, 1994, Cummings et al., 1996, Lisman et al., 2002). On the other hand, our previous work showed that a temporal offset of 15–20 ms in convergent input activity generated a spatial bias in axon branch stability, and furthermore that the temporal sequence of convergent input activity within this interval was sufficient to direct the spatial distribution of branch dynamics toward either rostral or caudal directions (Hiramoto and Cline, 2014). Whether graded NMDAR activity plays a role in generating the spatial asymmetry in the branch stability within the retinotopic map is unclear. As NMDAR activity controls the direction of the branch tip shifts in response to temporally offset visual inputs, it may also play a role in generating spatial asymmetry in branch stability. We asked whether the attenuation of NMDAR currents affects the spatial distribution of stable branches, or if it uniformly reduces branch stability in all regions of the arbor without spatial bias.

**Figure 4:**
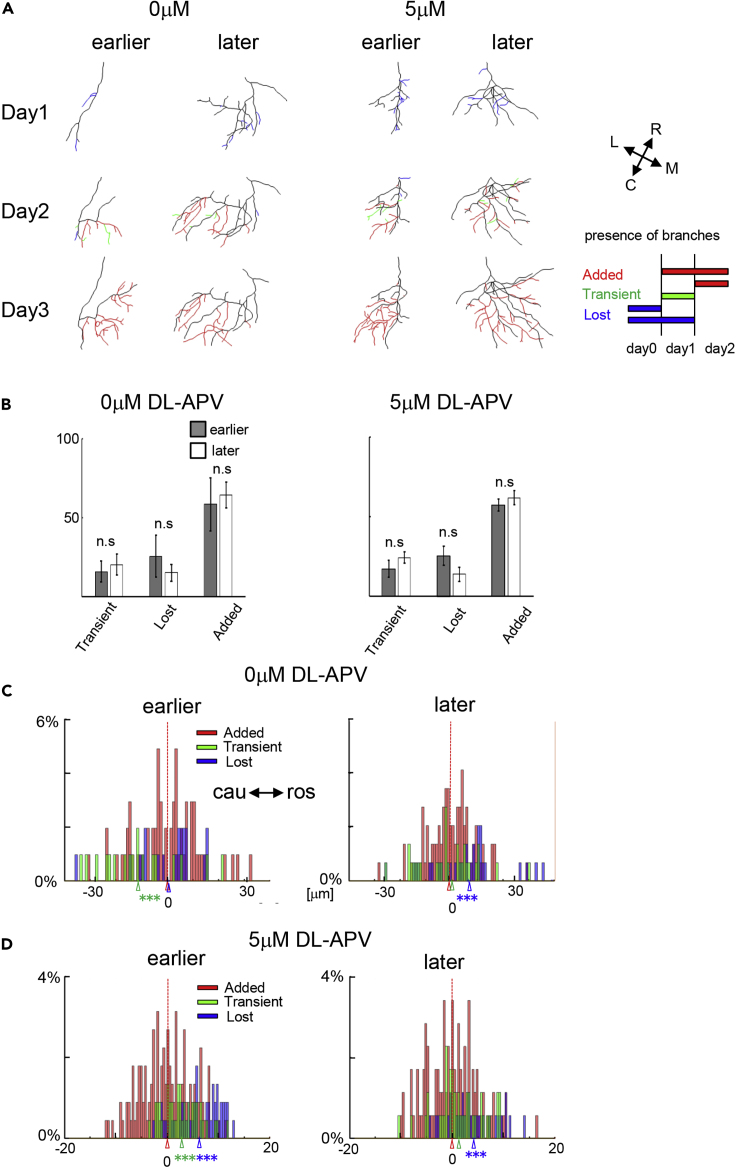
Graded NMDAR Function Alters the Spatial Distribution of Dynamic and Stable Branches (A) Representations of branch dynamics in axon arbors over 3 days (days 0, 1, 2) from earlier- or later-stimulated conditions in animals exposed to 0 μM APV or 5 μM DL-APV during the visual stimulation protocol. Reconstructions of axon arbors over 3 days show dynamic branches based on their presence at each time point. Branches are categorized and color coded according to the schematic shown in the lower right of the panel. Added branches, red; transient branches, green; lost branches, blue. The orientation of the arbors in the tectum is shown by the inset in the upper right panel. (B) The proportion of transient, lost, and added branches in axons from earlier- and later-stimulated conditions from animals exposed to 0 μM APV (left) or 5 μM DL-APV (right) was not significantly different. (C and D) Histograms of the spatial distribution of the branches along the rostrocaudal axis of the tectum in 0 and 5 μM DL-APV for the different dynamic branch categories: added (red), transient (green), lost (blue). x axis represents the locations of the branch tips along the rostrocaudal tectal axis, normalized to the distribution of added branch tips, with the median set as 0. ∗∗∗p < 0.001, Bootstrap test N = 10,000.

To address this question, we analyzed the branch dynamics in axons imaged in the experiment described above. Dynamic branches were categorized as added, transient, and lost, as shown in Figure 4A. Branches that were added during the imaging session and maintained through to the last image were classified as “added.” Branches that were added and subsequently lost during the imaging period were classified as “transient.” “Lost” branches are those that were initially present at the first image and were subsequently retracted during the imaging period. Both lost and transient branches are unstable branches. Importantly, the proportion of branches that display these dynamics was the same between the earlier- and later-stimulated conditions (Figure 4B, left). Branch stability between earlier- and later-stimulated conditions was not different in 5μM DL-APV either (Figure 4B, right). We plotted a histogram of the distribution of transient (green), added (red), and lost (blue) branches along the rostrocaudal axis with the center of all added branches set as the reference point (0). The statistical tests are compared with all the added branches using Bootstrap test (N = 10,000) (Figures 4C and 4D). In control conditions (0 μM APV), a temporal offset of 15 ms earlier or 15 ms later stimulation induced a caudal and rostral spatial bias in branch dynamics, respectively. In axons that were stimulated later than convergent inputs, more branches were lost in the rostral side of the arbor (Figure 4C, right “Lost”), producing a net caudal shift in branch tip positions. In the earlier-stimulated axons, more transient branches were observed in the caudal region of the arbor (Figure 4C, left, “Transient”), generating a net rostral shift in branch tip positions. However, in animals exposed to 5 μM APV during the visual stimulation protocol, in earlier-stimulated axons, the locations of the lost and transient branches were biased to the rostral axon territory side (Figure 4D left, “Lost,” “Transient”), indicating greater branch instability in the rostral territory of the arbor, generating a caudal shift in the arbor. In later-stimulated axons, the spatial distribution of the dynamic branches was similar between control and 5 μM APV (Figures 4C and 4D right). These data indicate a stronger driving force to shift branches toward caudal tectum in the earlier-stimulated axons when NMDAR is attenuated. These data further suggest that the inverted shift in the branch tip movement shown in Figure 3 arises from greater stability of newly added branches in the caudal region of the arbor combined with greater branch dynamics in the rostral region of earlier stimulated axons.

### Reversal of Directed Arbor Growth in Neighboring Sub-arbors

**Figure 5:**
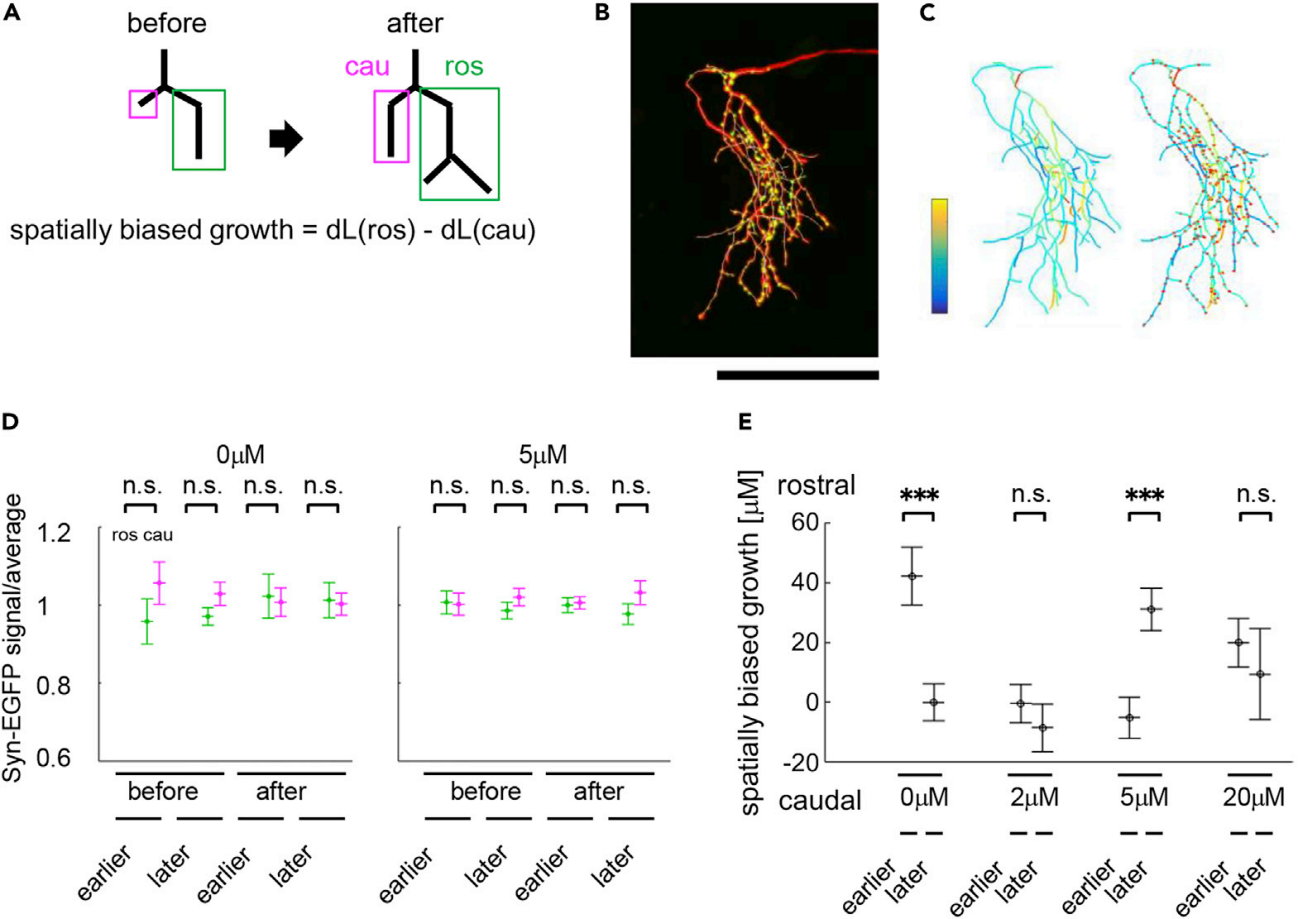
Reversal of Directed Arbor Growth in Neighboring Sub-arbors (A) Analysis of spatially biased growth of sub-arbors. Sub-arbors were defined as rostral or caudal according to the gravity center of the branch positions. Spatially biased growth was quantified as the difference in the change in branch length between the rostral and the caudal sub-arbors over the imaging interval. All branching points were analyzed, and the data were pooled (Figures S3 and S4). (B) An image of an axon expressing tdTomato and synaptophysin-EGFP to identify synaptic puncta. (C) Reconstruction of the axon in (B) with branches color-coded according to the intensity of the synaptophysin-EGFP signal. Red dots identify the puncta locations. Synaptophysin-EGFP intensity per branch length was measured and normalized to mean value across the axon. Right: overlay of puncta. (D) Plot of synaptophysin-EGFP intensity per unit branch length between neighboring rostral and caudal branches. Data are from animals treated with or without DL-APV and before and after 2 days of visual stimulation with the dt = ±15-ms protocol. No significant difference was detected. See Table S2 for data and p values. (E) Differences in branch lengths between the sibling sub-arbors across DL-APV doses for axons from earlier- or later-stimulated conditions. In controls, sub-arbors in earlier-stimulated axons showed a rostral spatial bias in sub-arbor growth, but in 5 μM APV, sub-arbors in later-stimulated axons showed a rostral growth bias. ∗∗∗p < 0.001. Bootstrap test, N = 10,000. (0 μM: earlier group: N = 100 branch points, 7 animals; later group: N = 132 branch points, 7 animals. 5 μM: earlier group: N = 213 branch points, 7 animals; later group: N = 120 branch points, 7 animals). See Table S2 for data and p values.

Axon arbors have many branch points, each of which gives rise to two sub-arbors (Figure 5A). A spatial shift in arbors could arise from a consistent bias in the growth between neighboring sub-arbors along the rostrocaudal tectal axis, as schematized in Figure 5A. In animals stimulated with sequential inputs, the changes in axon branch length did not appear uniform across branches in sub-arbors (Figures 1B and 1C), suggesting a spatial bias in the growth of rostral and caudal sub-arbors. To address this possibility, we first tested whether synaptic puncta were distributed evenly in the axon arbor by calculating the average synaptophysin-EGFP signal intensity in each segment between the branch points (Figures 5B and 5C). We found that the average intensity per branch length was not significantly different between the rostral and caudal branch segments (Figure 5D). Then we determined the branch lengths of the two sub-arbors for each branchpoint (Figures S3 and 5E). In controls, sub-arbors in earlier-stimulated axons showed a rostral spatial growth bias, but in 5 μM APV, this distribution in spatially biased arbor growth was reversed so that sub-arbors in later-stimulated axons showed a rostral growth bias (Figure 5E). No spatial bias in sub-arbor growth was seen in the intermediate APV concentration (2μM) or the higher concentration (20 μM). These data indicate that the spatial growth advantage in rostral sub-arbors under earlier-stimulated conditions would enhance synaptic input from these arbor regions.

### Asymmetric NMDAR Activity between Two Convergent Inputs Redirects Branch Growth

**Figure 6:**
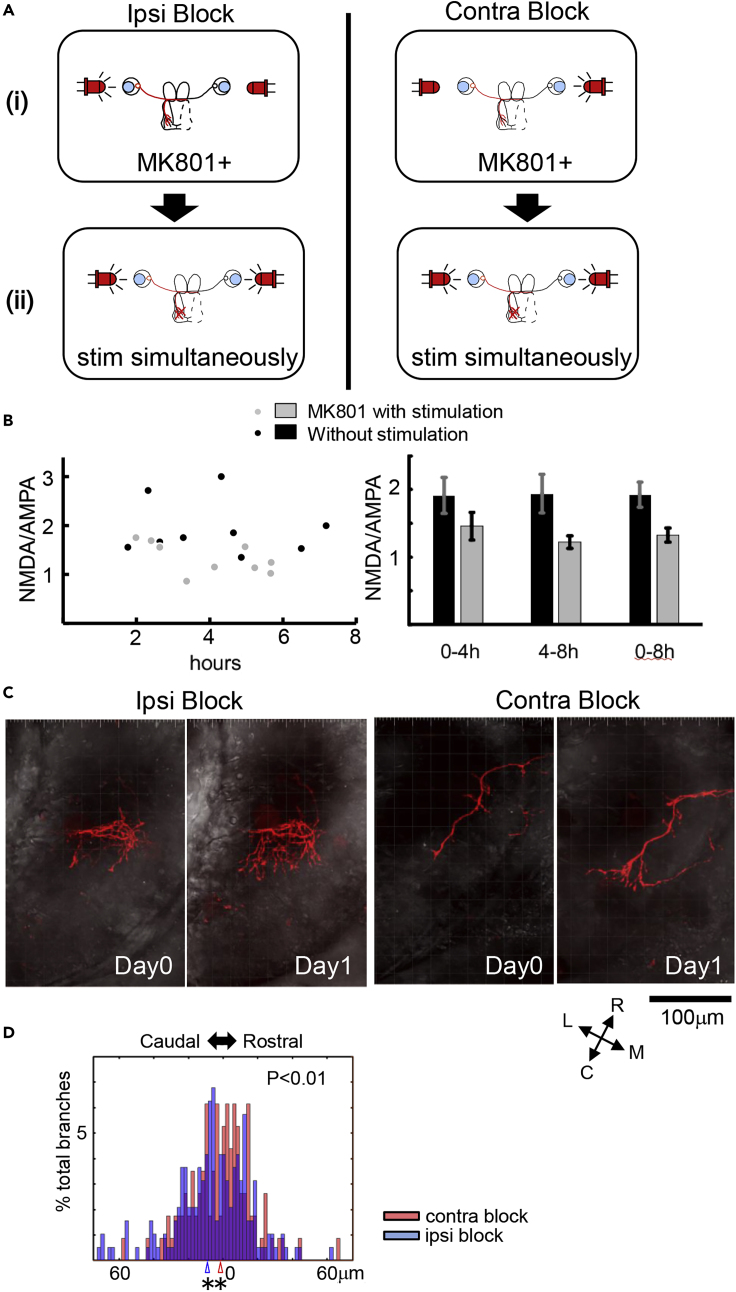
Asymmetric NMDAR Activity between Two Convergent Inputs Redirects Branch Growth (A) Schematic of the experimental design. Top: Animals with a dually innervated optic tectum and single labeled retinotectal axons were used. NMDAR activity in one retinal pathway, ipsilateral or contralateral to the tectum with the labeled axon, was attenuated by stimulating the eye with flickering LED in the presence of 2 μM MK801 for 15 min (IPSI Block and CONTRA Block, respectively). To prevent the activation of the other eye by the flicker, the other side was illuminated with constant light from the LED. Bottom: After washing out MK801, both eyes were stimulated simultaneously and branch dynamics were quantified using *in vivo* time-lapse imaging. (B) Left: Plot of NMDAR current normalized to AMPAR current recorded at specified times after MK801 treatment. Right: Plot of average NMDAR/AMPAR current ratios binned over time. (C) *In vivo* time-lapse confocal z series images of axon arbors imaged on Day 0 and Day 1 in animals under IPSI Block and CONTRA Block conditions superimposed on differential interference contrast images of the tectum. (D) Histograms of the amplitudes of branch tip movement toward the rostral or caudal tectal poles for axons imaged under IPSI (blue) and CONTRA (red) Block conditions. Bin = 2 μm. Bootstrap (N = 10,000) was used for statistics. ∗∗p < 0.01. (N (IPSI) = 144 branches in 6 axons, N (CONT) = 237 branches in 6 axons.

The inverted branch tip shift in 5 μM APV indicates that the amplitude of the NMDAR current is sufficient to control the direction of branch tip shift. This suggests that information required for the temporal to spatial transformation is encoded by the amplitude of NMDAR response in a manner such that a larger response drives a rostral shift in branches. Alternatively, the spatial information may not be mediated directly by the amplitude of the NMDAR signal and the NMDAR signal could modulate other signaling pathways that mediate the structural plasticity. To distinguish these alternatives, we tested whether decreasing of the strength of NMDAR activity in one of the convergent retinotectal pathways would produce a shift in the distribution of axon branches when both input pathways are stimulated simultaneously. To attenuate NMDAR responses in one of the two retinotectal pathways, we employed the use-dependent NMDAR antagonist MK801 (Figure 6). We treated animals with 2 μM MK801 and presented flickering on/off visual stimuli to one eye with an LED, whereas the other eye received continuous light on stimulus (Figure 6A), which prevents retinal activation (Hiramoto and Cline, 2014). After 15 min the drug was washed away. We measured the NMDAR- and AMPAR-mediated currents by whole-cell patch clamp at −60 and +55 mV using minimal stimulation (Wu et al., 1996) and determined the change in NMDAR responses normalized to AMPAR responses over periods up to 8 h after MK801 treatment. We observed an ~30% reduction of NMDAR current on the stimulated side with MK801 that persisted for up to 8 h (Figure 6B). To test whether this unilateral attenuation in synaptic NMDAR currents driven by one eye would affect the distribution of axon arbors in response to stimulation of the convergent retinal inputs, we imaged individual axons in animals in which both eyes were simultaneously stimulated by flickering light for 10 h (Figure 6C) and analyzed the axon branch tip shift. Under conditions in which retinotectal NMDAR synaptic currents were attenuated in the imaged axon (IPSI Block), branch tips shifted caudally compared with the branch shift under the CONTRA block condition in which NMDAR currents were attenuated at synapses activated by the other eye (Figure 6D). These data show that relative differences in NMDAR response amplitudes in convergent inputs are sufficient to induce shifts in branch tip positions.

## Discussion

The temporal sequence of neural activity is known to encode spatial information (Buzsaki and Tingley, 2018, Foster and Knierim, 2012); however, mechanisms that decode the temporal sequence of activity have not been reported. We took advantage of the experience-dependent plasticity of the visuotopic map in *Xenopus* optic tectum to analyze the transformation of the temporal sequence of RGC activity into the spatial order of axons in the tectum. Our results show that NMDAR activity translates the temporal sequence of afferent activity into the spatial representation of retinotectal axon arbors. NMDARs are recognized to play essential roles in several forms of plasticity operating over different timescales (Bittner et al., 2017, Buonomano and Maass, 2009, Constantine-Paton, 1990, Schiller et al., 2018), including roles in structural plasticity, where NMDARs are thought to stabilize coactive inputs and destabilize inputs that are not coactive (Munz et al., 2014, Rajan et al., 1999); however, the role of NMDARs in structural plasticity beyond this concept has been unclear. By quantifying the directional mobility of axon branches in the tectum in response to visual stimulation *in vivo* we show that the magnitude of NMDAR responses determines the direction of axon branch rearrangements along the rostrocaudal tectal axis, thereby governing the transformation of sequential temporal activity in RGC axons into the spatial order of axon position within the visuotopic map. We further demonstrate that the magnitude of NMDAR activity controls the transformation of temporal to spatial information by regulating the distribution of dynamic axon branches, such that decreasing the magnitude of NMDAR activity by half reversed the experience-dependent redistribution of stable and dynamic branches along the rostrocaudal tectal axis and biased the directional growth of axons in response to sequential patterns of visual input. Using MK801, we further demonstrate that asymmetric NMDAR activity between convergent inputs is sufficient to control their positioning along the rostrocaudal tectal axis. We note that the MK801 likely underestimates this outcome because newly formed synapses are not influenced by this manipulation. Together, these data indicate a role for NMDARs in detecting the temporal sequence of afferent input and converting this temporal information into the spatial distribution of afferent inputs in the target circuit by structural plasticity-based branch dynamics. This general concept of NMDAR function in temporal to spatial transformation may extend beyond sensory map plasticity to broader functions, such as hippocampal place cell formation and plasticity (Bittner et al., 2015, Bittner et al., 2017), where it has been suggested that hippocampal place cells extract spatial information about the environment an animal has recently navigated from the temporal sequence of their input activity (Burgess and O'Keefe, 2011, Lisman and Buzsaki, 2008). Importantly, place fields are constantly updated to incorporate information from new environments and to accommodate requirements of spatial memory (Bittner et al., 2017), potentially analogous to the visual-experience-dependent updating of visual projections (Cline, 1998, Espinosa and Stryker, 2012, Stryker and Lowel, 2018). It would be interesting to see if modifying the temporal sequence of afferent activity to place cells, for instance, in a virtual reality setting, reorganizes the sequential order of place cell responses in the hippocampus analogous to our results in tectum. Furthermore, our observations that the temporal to spatial component of the STS rule in the retinotopic projection can be inverted by attenuating NMDAR response amplitude suggest that the temporal to spatial transformation may be modulated in other circuits by mechanisms that affect NMDAR downstream signaling.

Numerous studies have shown that NMDARs are required for topographic map formation and plasticity in diverse sensory systems across vertebrate species. This together with studies showing that experience- and NMDAR-dependent topographic map refinement was mediated by dynamic rearrangements of axonal branches contributing to redistribution of axonal projections (Cline, 1991, Cline et al., 1987, Erzurumlu and Gaspar, 2012, Iwasato et al., 2000, Rajan et al., 1999, Ruthazer et al., 2003, Witte et al., 1996) suggested that NMDAR-dependent synaptic strengthening and weakening contributed to sensory system structural plasticity. Identifying the role of NMDAR in the STS transformation that occurs with experience-dependent visuotopic map plasticity suggests that NMDARs affect sensory map organization by decoding temporal information in retinal inputs. The biophysical properties of NMDARs indicate that they can detect convergent input activity firing in a time window of hundreds of milliseconds, consistent with their role in development and plasticity of organized sensory projections (Constantine-Paton et al., 1990). The potential significance of these features with respect to detecting and translating the temporal sequence of activity into spatial information in topographic maps was recently recognized based on experiments showing that anterior to posterior visual motion stimulation in optic flow was required to maintain retinotopic maps and that posterior to anterior motion stimulus failed to organize the topographic projection (Hiramoto and Cline, 2014). Recent work demonstrating that the temporal to nasal directional orientation of spontaneous retinal wave activity in mammals (Stafford et al., 2009) is propagated throughout the visual system during early postnatal development (Ackman et al., 2012) suggests that a similar NMDAR-mediated transformation of temporal information into spatial maps occurs widely in the development and tuning of sensory maps.

**Figure 7:**
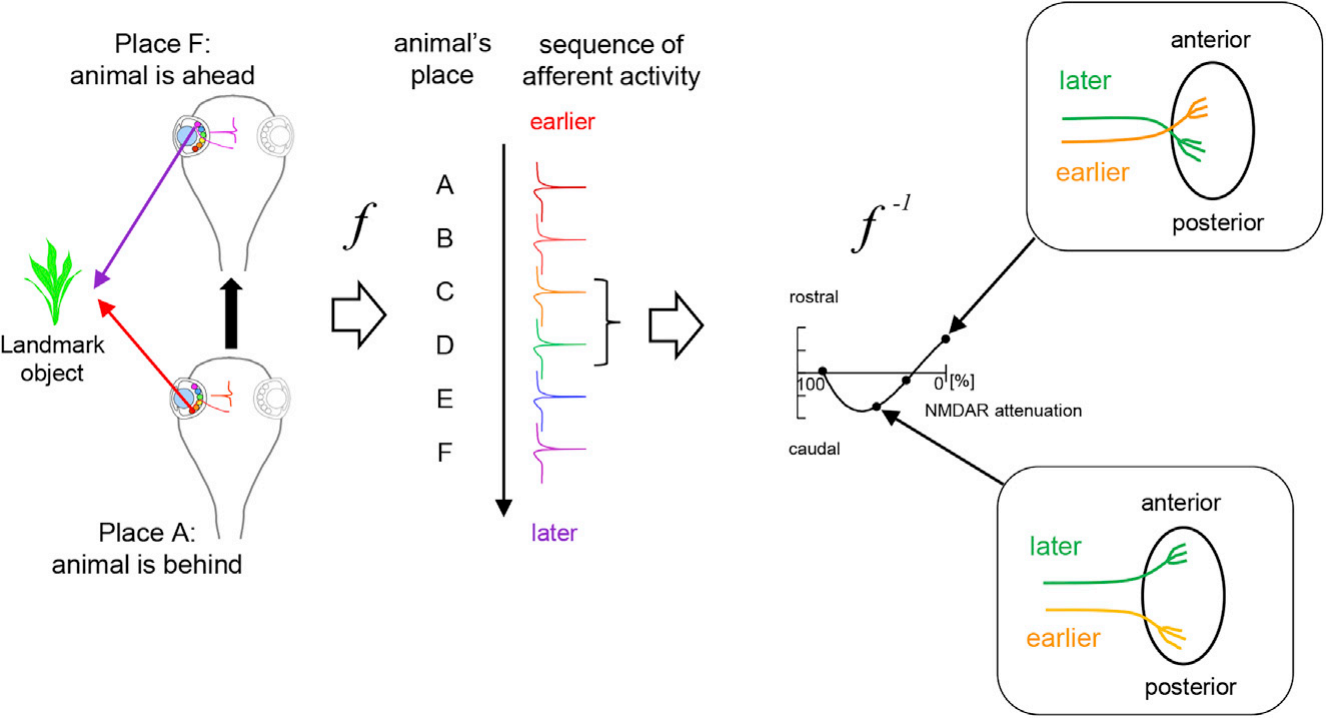
Schematic Model of the Role of NMDAR in Circuit Patterning As an animal moves past a landmark object, from position A, behind the landmark, to position F, in front of the landmark, RGCs from temporal to nasal positions are sequentially activated (left). Consequently, as the animal moves from position A to F, information about the landmark object in the RGCs' receptive fields is transcribed into the temporal sequence of the RGC activity (middle). This sequential activity pattern in retinotectal afferents is transformed into a visuotopic spatial map (*f*^*−1*^, right, top), but the temporal to spatial transformation is inverted when NMDAR signaling is decreased (right, bottom).

STS is a circuit-based mechanism that conveys spatial information in the form of the temporal sequence of convergent afferent activity and then tectal neurons extract a spatial representation from the temporal sequence of input activity, based on a forward transformation of information from spatial-to-temporal modalities (Figure 7 left) and an inverse transformation from temporal-to-spatial modalities (Figure 7 right). Our data indicate that this decoding mechanism retrieves spatial information from sequential afferent activity based on NMDAR activity. The unique biophysical properties of NMDAR activity and the protracted time course of its calcium conductance and downstream biochemical signaling enable NMDAR-mediated synaptic currents to encode the temporal history of synaptic inputs (Buonomano and Maass, 2009). In addition, the outcome of NMDAR-mediated synaptic plasticity depends on the response amplitude (Bear, 2003, Cummings et al., 1996), likely as a result of differences in downstream signaling pathways activated (Lisman et al., 2002). However, compared with synaptic plasticity, our understanding of the potential role of NMDAR in structural circuit reorganization is limited, and most studies assume that circuit reorganization occurs by synaptic plasticity-based mechanisms that operate over relatively short timescales. Our studies suggest that retinal axons converge on tectal cells and that a retrograde signal from the tectal cell dendrites/postsynaptic sites to retinal axon branches triggers changes in branch dynamics. It is possible that different amplitudes of tectal neuronal NMDAR responses activate CaMKII or calcineurin/protein phosphatase1 (Morishita et al., 2001, Xia and Storm, 2005) and induce different axon branch dynamics through distinct retrograde signals. In principle, presynaptic NMDAR could detect the temporal sequence of spikes in the neighboring RGC axons; however, electron microscopy studies in *Xenopus* tectum have not identified axo-axonal synapses (Li et al., 2011, Udin and Fawcett, 1988) and the axo-axonal interaction model is not consistent with the results of the MK801 experiments (Figure 6), showing that decreasing NMDAR activity in one of the two convergent pathways interferes with the directional branch dynamics. NMDARs are also required for circuit-based plasticity following exposure to different patterns of visual inputs (Pratt et al., 2008); however, the downstream plasticity mechanisms underlying this plasticity could be based on changes in synaptic strengths within the network, without involving structural plasticity of axonal connections induced by the temporal sequence of visual stimuli. The simplest interpretation of our results is that postsynaptic NMDARs detect convergent inputs that are sequentially active over intervals of tens of milliseconds (Shouval et al., 2002) and that this leads to iterative changes in axon branch dynamics and synaptic contacts with the circuit over a timescale of hours to days; however, direct support for this model requires the capacity to visualize the spatial and temporal properties of signaling cascades downstream of NMDARs. Alternatively, the temporal sequence of convergent inputs may be detected by other mechanisms and this information is then transformed into the different amplitudes of NMDAR signals.

Visual experience-dependent plasticity of visuotopic spatial maps organizes inputs based on the spatial arrangement of the afferents' receptive field, not the physical location of the afferents' soma, in contrast to chemospecific maps formed by molecular cues. Consequently, plasticity of visuotopic spatial maps functions to maintain calibration between the visual field and target area. Like the human face, the position of the eyes in tadpoles and frogs varies over time, requiring tuning mechanisms for calibration. The STS mechanism ensures that the visuotopic spatial map is represented across the anterior to posterior tectal extent (Figures 1 and 3), by regulating the growth of neighboring branches (Figure 5) and the spatial distribution of the lost or added branches (Figure 4). Together, these effects on branch dynamics and stability generate global rearrangements of retinotectal connections.

### Limitations of the Study

Because MK801 is a use-dependent NMDAR blocker, manipulating NMDARs with MK801 in the experiment shown in Figure 6 is limited to branches with pre-existing synapses. The difference in the growth between the two groups is likely to be initial growth after the start of stimulation. It is likely that part of the difference is masked by the variance of the subsequent growth of new branches. This may underestimate the difference.

### Resource Availability

#### Lead Contact

For correspondence or materials, contact Hollis Cline (cline@scripps.edu).

#### Materials Availability

This study did not generate new unique reagents.

#### Data and Code Availability

The datasets generated during this study are available at Hiramoto, Masaki (2020), “STS transformation-2”, Mendeley Data, V1, http://dx.doi.org/10.17632/bbth3twmfj.1. This is the link to the data: https://data.mendeley.com/datasets/bbth3twmfj/draft?a=63d4616a-1b7a-4ac3-b5ee-87110ddf1240.

## Methods

All methods can be found in the accompanying Transparent Methods supplemental file.

## Supplemental Information

Document S1. Transparent Methods, Figures S1–S3, and Tables S1 and S2
