# Supplementary material for: NMDARs Translate Sequential Temporal Information into Spatial Maps
Source: iScience. 2020 May 1;23(6):101130. doi: 10.1016/j.isci.2020.101130 (PMC7262552; doi:10.1016/j.isci.2020.101130)
Supplement: Document S1. Transparent Methods, Figures S1–S3, and Tables S1 and S2 [file mmc1.pdf]

iScience, Volume *23*

Supplemental Information

NMDARs Translate Sequential Temporal Information into Spatial Maps

Masaki Hiramoto and Hollis T. Cline

Supplemental Data

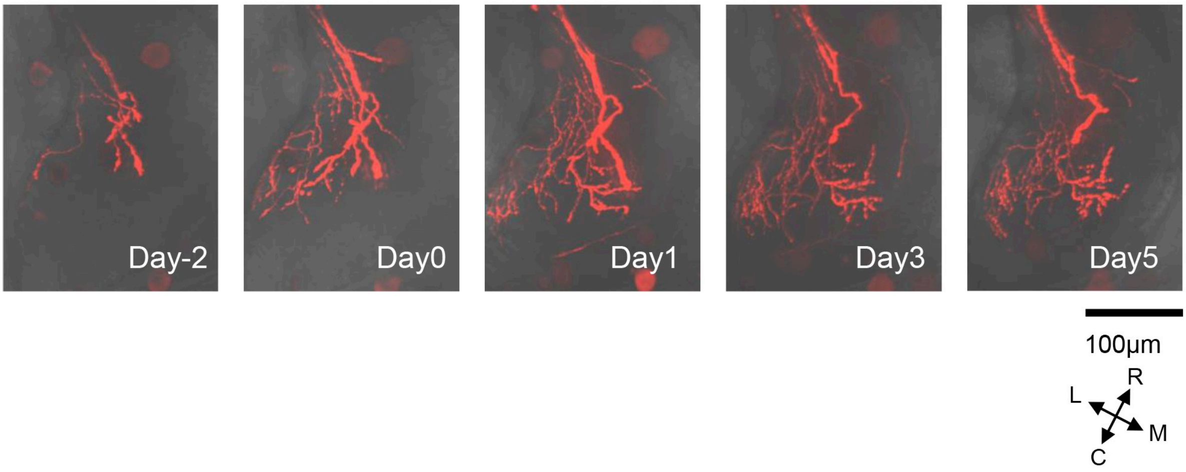

Figure S1. Global movement of branches induced by sequentially ordered inputs. Related to Figure 1.

Shift in ipsilateral retinal axon arbors over 5 days of sequential stimulation of convergent retinotectal inputs. Stimulus was provided 15ms earlier than the contralateral side. Time-lapse confocal Z series images of several RGCs from the left eye that were imaged over 7 days, with sequential stimulation starting after the image was collected on Day 0. The animals were raised in dark until day 0. The eye with the labeled axon was stimulated for 10h per day from Day 0 until Day 5. Labeling several axons facilitates visualization of the drastic shift in branches in the population of axons. Note that many axons turned and extended toward rostral tectum after the sequential stimulation started.

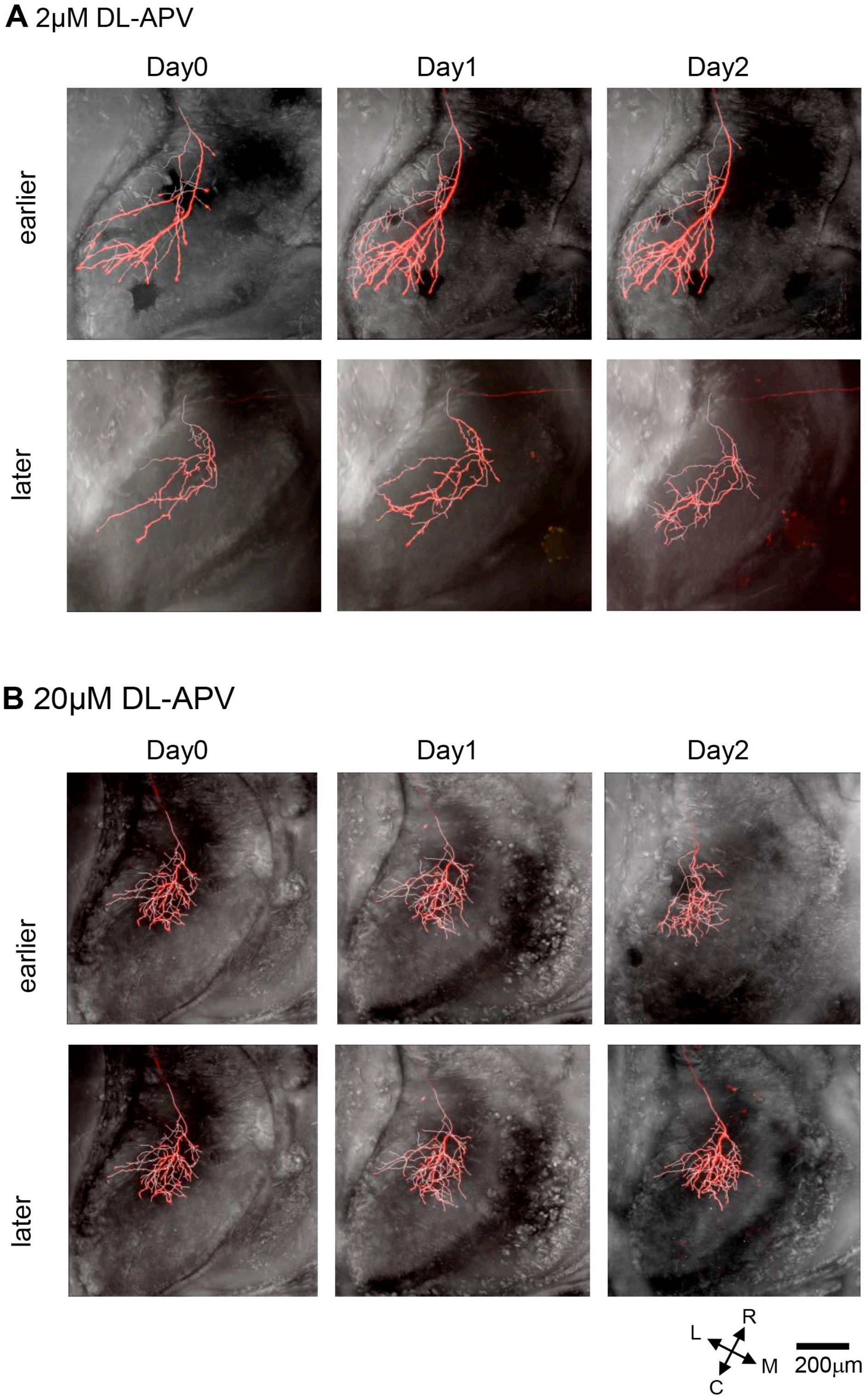

Figure S2. Branch shifts in various concentration of DL-APV. Related to Figure 3.

A, B Example images of experiments in Fig. 3, in which different concentrations (A: 2uM and B: 20uM) of DL-APV were used. Stimulation started after imaging at day 0. Inter-stimulus interval is 15ms.

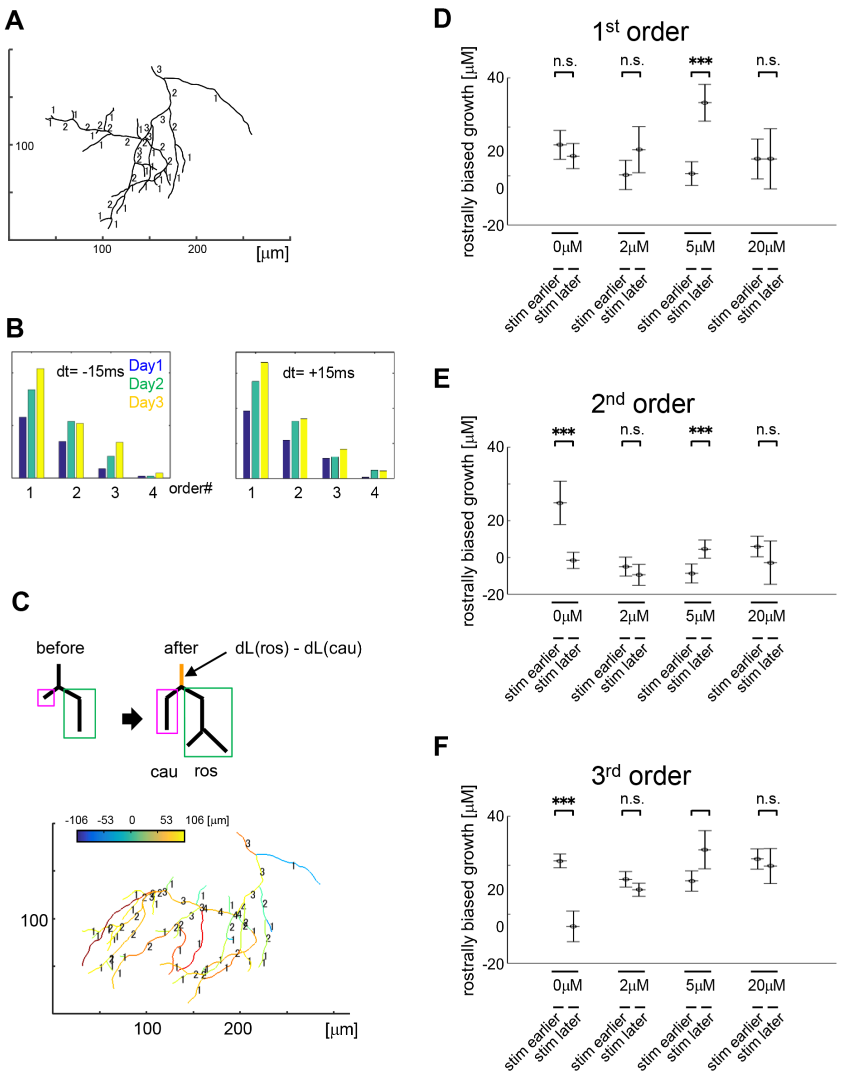

Figure S3. Spatially-biased growth of sub-arbors at each branch order analyzed using the Strahler method. Related to Figure 5.

A. An example of branch order assignment in an axon arbor. The order was determined by the Strahler method (Strahler, 1952). B. Histograms of branch order of the converged axons. The axons are stimulated 15 earlier (left) or 15ms later (right) than the other group of axons. Day1: before stimulation. Day2 and Day3: stimulated for 1day or 2 days, respectively. The distribution of the branch order is not significantly different between earlier and later groups. C. Schematic of analysis of rostral and caudal branch dynamics. Rostral and caudal branches are shown in color coded boxes (top). Example of an arbor stimulated 15ms later than convergent inputs (bottom). The color of the branch segment indicates changes of the total branches sprouting from the colored segment. D. Growth of the rostral and the caudal sub-arbors of each branching order was compared. The N-th order indicates comparison of sub-arbors bearing from N+1th order branching point. In most of the cells, 4th order is the highest order.

Z (n)	0μM		2μM		5μM		20μM	
Axon ID	earlier	later	earlier	later	earlier	later	earlier	later
1	-0.56 (12)	-0.17 (39)	-0.47 (39)	-0.065 (37)	-0.11 (28)	-0.26 (26)	-0.025 (133)	-0.82 (5)
2	-0.26 (26)	-0.17 (27)	-0.56 (10)	-0.11 (36)	-0.057 (58)	-0.11 (21)	-0.26 (25)	-0.82 (7)
3	-1.2 (8)	-0.26 (37)	-0.076 (108)	-0.22 (21)	-0.32 (17)	-0.19 (40)	-0.12 (142)	NaN (3)
4	-Inf (5)	-0.81 (8)	-0.78 (8)	-0.080 (96)	-0.32 (31)	-0.19 (29)	-0.40 (37)	-0.17 (34)
5	-0.32 (21)	-0.29 (6)	-0.025 (131)	-0.78 (25)	-0.14 (54)	-0.22 (38)	-0.090 (83)	-0.074 (45)
6	-0.066 (39)	-0.043 (66)	-0.13 (25)		-0.17 (52)	-0.051 (54)		-0.18 (18)
7	-0.40 (33)	-0.41 (36)			-0.14 (55)	-0.80 (13)		-1.2 (14)
8								-0.088 (151)
Random [y/n]	y	y	y	y	y	y	y	y

Table S1

Wald–Wolfowitz runs test to evaluate the independence of the direction of branch tip movement. Related to Figures 1, S3.

Tables of the z values calculated by the Wald–Wolfowitz runs test are shown. The shift is not random when |z| is larger than 1.96 [=Z(1-alpha/2): alpha= 0.05]. The branch movement data were arranged so that the branch #N + 1 is the branch whose tip is closest to the branch #N before stimulation. The first branch in the series was chosen so that the variance of the intertip distances was the smallest. To apply the Wald–Wolfowitz runs test, the directionality of the shift along the rostrocaudal axis was analyzed. The data show that the branch tip movements were random for all axons.

(for Fig. 5D)

DL-APV	0μM		5μM	
Stim order	-15ms	+15mw	-15ms	+15ms
Before train	0.11 (46,49 [7])	0.06 (89,98 [7])	0.47 (114,121 [6])	0.14 (153,160 [6])
After train	0.41 (56,58 [7])	0.43 (97,105 [7])	0.40 (77,79 [7])	0.08 (154,157 [7])

(for Fig. 5E)

DL-APV	0μM	2μM	5μM	20μM
	2.0e-4 (100 [7], 132 [7])	0.20 (185 [6], 106 [5])	1.0e-4 (213 [7], 120 [7])	0.27 (206 [5], 69 [8])

Table S2.

P values and the number of branches and animals analyzed in the graphs in Fig. 5D and E. Related to Figures 5D, E.

Format, 5D (top): P (N branches in 'earlier' group [N animals], N branches in 'later' group [N animals]).

Format, 5E (bottom): P (N branch points in 'earlier' group [N animals], N branch points in 'later' group [N animals])

Transparent Methods

All experimental protocols were approved by The Scripps Research Institute Institutional Animal Care and Use Committee and complied with the guidelines established in the Public Health Service Guide for the Care and Use of Laboratory Animals.

Tadpole Preparation. Albino *Xenopus* laevis tadpoles were obtained either by mating frogs in our colony or purchased from Xenopus Express Inc. All tadpoles were reared in an incubator at 22 °C with a 12 h light/12 h dark cycle until the experiments started. For imaging experiments in dually innervated tecta, RGCs were sparsely labeled and the next day the tectal lobe contralateral to the labeled eye was ablated (Ruthazer et al., 2003). For labeling, RGCs in stage 41 tadpoles were sparsely transfected with plasmids encoding tdTomato and Synaptophysin-GFP. Animals were anesthetized in 0.01% MS-222 solution and placed on an electrically grounded moist kimwipe. Two plasmids, pCMV:GAL4/UAS:TdTomato and pUAS:Synaptophysin-GFP (final concentrations, 0.5 μg/μL and 0.25 μg/μL, respectively), were mixed and pressure injected from a micropipette inserted between the retina and lens, using a picospritzer (Picospritzer II, General Valve Corporation). Fast Green (0.01%) was added to the DNA solution to monitor injection into the eye. The pipette tip was placed close to the center of the retina, and cells were electroporated by applying a single 37-V pulse of 1.6 ms. In the ablation of the tectal lobe, the optic nerves were cut at the optic chiasm to synchronize retinal innervation of the remaining tectal lobe. For each experiment, animals were screened 3 d after surgery to identify those with well labeled retinotectal axons. Tadpoles were kept in the dark after tectal ablation until stage 48 to prevent visual experience dependent effects on retinal axon arbors.

Visual Stimulation Protocols. During visual stimulation, the animals were positioned between the edges of the two acrylic plates. The edges were shaped so that they snuggly hold the animals. An LED is attached to the other edge of the acrylic plate. The bottom and top of the chamber are covered with a mesh and a cover slip, respectively. For experiments in animals with dually innervated tecta, both eyes were stimulated with light (343 cd/m2) from an LED (OptoSupply, OSHR5161A-QR, peak at 625 nm) delivered through a clear plastic plate. The edge of the plastic plate facing the animal was coated with white paint for even illumination of only one side of the eyes. Since evoked EPSCs occur when the light intensity changes and not when the light remains on, one eye can be selectively stimulated by presenting dimming light to one eye and persistent light on to other eye (Hiramoto and Cline, 2014). Some animals were treated with the NMDAR blocker DL-APV (2~20 μM) in rearing solution throughout the 10 hour stimulation protocol. Some animals were treated with MK801 during visual stimulation. Different use-dependent NMDAR inhibition between left and right visual pathways was generated by presenting flickering light to one side and persistent

light to other side in the presence of MK801. Animals were first stimulated in Steinberg and 2μM MK801 was introduced after 5 min. After that, dimming light was presented at 1/5.5Hz. The eyes were stimulated for 15 min. Steinberg solution was changed three times to remove MK801 in the bath. After 15min, Steinberg solution was replaced, and both eyes were stimulated with 11 Hz dimming light (100% to 19%) for 5 hours.

Electrophysiology. Stage 47/48 tadpoles were anesthetized in 0.02% MS222 and stabilized on Sylgard with dissecting pins. To record evoked responses, the skin over the tectum was removed, and dissected in extracellular saline [115 mM NaCl, 4 mM KCl, 3 mM CaCl2, 3 mM MgCl2, 5 mM Hepes, 10 μM Glycine, 10 mM Glucose (pH 7.2)]. A step of -5mV was applied to check consistency of access resistance. Neurons had series resistances <100MΩ. Electrophysiological whole-cell recordings were taken from tectal neurons, using glass micropipettes(8–12 MΩ) filled with intracellular saline (containing, in mM:100 K-gluconate, 8 KCl, 5 NaCl, 1.5 MgCl, 20 HEPES, 10 EGTA, 2ATP, 0.3 GTP; pH 7.2, osm 255)(Aizenman and Cline, 2007). To measure the NMDAR and AMPAR currents and AMPA/NMDA ratio, optic nerves were stimulated with a bipolar stimulating electrode (FHC) using minimal stimulation and tectal responses were recorded at -60mV and +40mV. For data presented in Figure 2, NMDAR-mediated currents were measured between 20-30 ms after the onset of the current (Wu et al., 1996). For data presented in Figure 6, the peak AMPA response (measured between +/-5ms of the peak current) was used to calculate the AMPAR component and the amplitude between 20 and 30 ms after response onset was used to calculate the NMDAR component. The AMPAR component decays to 10% of the peak value in this time window (Aizenman and Cline, 2007). Recordings were performed with an Axopatch-200B amplifier and digitized using pClamp-8 software and a Digidata-1322A A/D-board (Axon Instruments). To measure attenuation of NMDAR current by MK801, the tadpoles were placed in Steinberg's solution containing 2μM MK801. The eyes were presented with flickering light on one side and persistent light on the other side. After 15min, LEDs were turned off and Steinberg's solution was changed three times. Animals were dissected at various time up to 7.5 hours and AMPAR- and NMDAR-mediated currents were recorded from both tectal lobes. Experiments and analysis were conducted blind to treatment.

In Vivo Time-Lapse Confocal Imaging and Analysis. Confocal images of retinotectal axons, labeled with cytosolic tdTomato and Synaptophysin-GFP, were collected with a PerkinElmer Ultraview Vox spinning disk confocal mounted on a Nikon FN1microscope with a 25×/N.A. 1.10 objective. The axon arbor morphology was reconstructed in the Filament tracing mode using IMARIS software. Branches longer than 2 μm were included. Images were collected once a day over 4 d before and after the visual stimulation sessions. The axon arbor morphology was reconstructed using IMARIS software. For analysis of the shift in branch tip positions, the filament information was

exported to MATLAB and analyzed. The traced filaments are aligned by an iterative closest point algorithm. Positional data from all corresponding branches were used for the alignment of the two axons. The shift of the branch tip position was calculated by subtracting the branch point to branch tip vectors before stimulation from the vector after the stimulation. The bias in the branch shift was analyzed by bootstrapping (10,000 repeats). Dynamic branches were categorized as added, transient, or lost (Ruthazer et al., 2003) as follows: Added branches are branches that emerged and persisted to the end of the imaging period. Branches that were added and subsequently lost were categorized as transient. Lost branches were present initially and retracted completely during the imaging period. The positions of the branches are measured at the last time point except for lost branches. The lost and transient branches are measured at the earliest time point when they exist. The spatial bias of the dynamic branches was analyzed by comparing the mean change in branch tip position with the center of mass of all added branches.

Statistical analysis. Bootstrap (N=10,000) was used to generate the statistic distribution of the difference of the means of the two groups of the RGCs. We used bootstrapping instead of a statistic test that assumes normal distribution because at least two mechanisms contribute to the branch dynamics: the rostrocaudal shift by STS and the segregation of the two groups of RGCs. In such a case, the driving force for the segregation skews the distribution in the overlapping area. Data are considered significantly different when p values are less than 0.05. Experiments and analysis were performed blind to the experimental conditions.

Data Availability. The data that support the findings of this study are available from the corresponding author upon reasonable request.

Supplemental References

Aizenman, C.D., and Cline, H.T. (2007). Enhanced visual activity in vivo forms nascent synapses in the developing retinotectal projection. J Neurophysiol *97*, 2949-2957.

Hiramoto, M., and Cline, H.T. (2014). Optic flow instructs retinotopic map formation through a spatial to temporal to spatial transformation of visual information. Proc Natl Acad Sci U S A *111*, E5105-5113.

Ruthazer, E.S., Akerman, C.J., and Cline, H.T. (2003). Control of axon branch dynamics by correlated activity in vivo. Science *301*, 66-70.

Strahler, A.N. (1952). Hypsometric analysis of erosional topograph. Bull geol Soc Am *63*, 1171-1182.

Wu, G., Malinow, R., and Cline, H.T. (1996). Maturation of a central glutamatergic synapse. Science *274*, 972-976.
